# Foundational Reading Knowledge of Teachers of Students With IDD: Examining Experience, Degree and Time Use

**DOI:** 10.1111/jir.70041

**Published:** 2025-09-04

**Authors:** Esther R. Lindström, Kimberly A. McFadden, Qiong Fu, Molly J. Ruiz

**Affiliations:** ^1^ University of Illinois Chicago Chicago Illinois USA; ^2^ Lehigh University Bethlehem Pennsylvania USA

**Keywords:** intellectual disability, reading, special education, teacher knowledge

## Abstract

**Background:**

Special education teachers require foundational reading content knowledge (e.g., phonemic awareness, phonics) to teach early reading skills. Though many measures have been developed to measure such knowledge, none have examined item‐level differences related to teacher characteristics (i.e., experience, degree and instructional time use).

**Method:**

In this study, we examined the psychometric properties of the 20‐item *Teacher Knowledge Assessment: Structure of Language* scale using data from 337 special education teachers providing reading instruction to students with intellectual and developmental disabilities in the United States.

**Results:**

Out of 20 possible total points for correctly answered items, the average score was 13.2 (SD = 3.5). We conducted Rasch analysis and dropped two misfitting items, resulting in 18 items on the scale (M = 11.9; SD = 3.3). Using dichotomous teachers' years of experience (≤ 5 years vs. > 5 years), education level (bachelor's or below vs. advanced) and self‐reported time teaching phonics and phonemic awareness (≤ 20% vs. > 20%) as focal variables, we conducted differential item functioning (DIF) analyses as part of the Rasch analysis. A greater number of items showed DIF for teacher experience or instructional time use (8 items each) than for degree (3 items), with easier and harder items identified for each subgroup.

**Conclusions:**

These results evince inconsistencies in teachers' acquired foundational reading knowledge based on experience, degree and instructional time use. Structured literacy standards for teacher preparation programmes and in‐service training initiatives may provide the means to address gaps in teachers' knowledge.

## 1 Introduction

Elementary special education teachers primarily working with students with intellectual and developmental disabilities (IDD) require a broad range of knowledge and skills to provide effective instruction (Brownell et al. 2020). In this study, we use the term IDD to refer to eligibility for special education services under various disability classifications (e.g., intellectual disability, autism, multiple disabilities and developmental delay), encompassing a range of support needs. As many students with IDD are educated in self‐contained, multigrade special education classrooms (Tiernan et al. 2020; Williamson et al. 2020), their teachers require content knowledge spanning multiple grade levels and content areas (e.g., reading and mathematics; Brownell et al. 2020). Teachers' knowledge of foundational reading content (e.g., phonemic awareness and phonics), aligned with principles of structured literacy, is of particular concern with regard to this population of students (Castles et al. 2018; Moats 2009). Structured literacy principles are those drawn from a large base of empirical evidence, often referred to as the science of reading.

Methods of word reading instruction are one area of focus within the science of reading, and for the general population (National Reading Panel [NRP] 2000), as well as for students with IDD (e.g., Sermier Dessemontet et al. 2019), evidence suggests that a code‐based approach to word reading instruction is more effective and efficient. Code‐based instruction involves teaching students to apply knowledge of the relationships between print and speech sounds (i.e., grapheme–phoneme correspondences) to decode words. However, whole‐word approaches to reading have been emphasised for students with IDD in the past (e.g., Browder et al. 2006; Bruni and Hixson 2017). The whole‐word approach to teaching word reading circumvents teaching grapheme–phoneme correspondences and instead focuses on memorisation of words as whole units. Though students can memorise words, code‐based approaches to word reading instruction are more transferable (e.g., Sermier Dessemontet et al. 2019). That is, students can apply knowledge of letter–sound correspondences to read words that have not been explicitly taught, making the code‐based approach preferable. Because of the historic focus on whole‐word instruction for students with IDD, it is possible that this subset of special education teachers has not been adequately trained to provide code‐based foundational reading instruction (Sermier Dessemontet et al. 2019). Prior research on preparing teachers to teach reading to students with extensive support needs identified several issues, including a lack of focus on code‐based approaches to word reading instruction and low expectations for literacy achievement for students (Copeland et al. 2011). Evaluating foundational reading knowledge across a national sample of special education teachers of students with IDD (henceforth referred to as SET‐IDDs) can provide a preliminary indication of how this knowledge aligns with research supporting code‐based approaches to reading instruction.

Though they do not directly measure teachers' knowledge, descriptive studies focused on reading instruction for elementary students with IDD raise additional concern about teachers' preparation and practices. An analysis of individualised education programmes for students with extensive support needs indicates an instructional focus on a narrow scope of foundational reading skills and minimal access to grade‐level material (Restorff and Abery 2013; Zagona et al. 2024). Similarly, observation studies examining business‐as‐usual reading instruction for students with IDD (i.e., Lindström and Lemons 2021; Sermier Dessemontet et al. 2022) indicate inconsistencies in the use of evidence‐based practices across teachers. Sermier Dessemontet et al. (2022) reported only about half of the Swiss teachers they observed providing systematic phonics instruction. In the United States, Lindström and Lemons (2021) noted discrepancies in teachers' use of evidence‐based practices (i.e., code‐based approaches vs. whole‐word approaches) for word reading instruction for primary students with IDD. Together, these findings suggest misalignment between research and practice and invite further investigation of SET‐IDDs' foundational reading content knowledge.

For this study, we selected the *Teacher Knowledge Assessment: Structure of Language* (TKA:SOL; Bos et al. 2001) among several instruments designed for this purpose (e.g., Binks‐Cantrell et al. 2012; Hall et al. 2024). This is the first study to evaluate the psychometric properties of a foundational reading knowledge tool using Rasch modelling (Rasch 1960) with differential item functioning (DIF) analyses to examine item‐level differences across subgroups of teachers (i.e., experience, degree and instructional time use). The following paragraphs review previous studies examining the psychometric properties of the TKA:SOL using various methods.

### 1.1 Psychometric Properties of Foundational Reading Knowledge Measures

Standards for measurement in education research highlight the importance of accumulating sources of validity evidence (i.e., test content, response processes, internal structure [including reliability], relations to other variables and consequences) when developing assessments or using existing instruments with a new population (American Educational Research Association [AERA] et al. 2014; Bandalos 2018). Strong evidence of validity (i.e., the degree to which assessments measure what they are intended to) and reliability (i.e., consistency of results obtained using the measure) increase confidence in findings obtained using the measure (Bandalos 2018). Test developers may choose from two approaches to measuring validity and reliability: classical test theory (CTT) and item‐response theory (IRT). The 1‐parameter logistic (1‐PL) IRT model, also known as the Rasch model, offers advantages over the CTT approach in that it focuses on item‐level data, offering valuable information about the relative difficulty of items and differences in item functioning between subgroups of respondents (Bandalos 2018). In many cases, researchers investigating teachers' foundational reading knowledge have developed their own instruments or modified existing instruments and reported results with limited or no examination of their psychometric properties (Hudson et al. 2021), calling attention to the need for further work in refining available instruments, such as the TKA:SOL. In the subset of studies that have collected sources of validity evidence for this measure, researchers have primarily used CTT approaches (e.g., Binks‐Cantrell et al. 2012; Hall et al. 2024), and few have reported using IRT (e.g., Beachy et al. 2023). Results have indicated good or acceptable psychometric properties.

We selected the TKA:SOL due to its focus on skills in need of prioritisation in research and instruction for elementary students with IDD (i.e., phonemic awareness, phonics). Additionally, because the TKA:SOL is approximately half the length of other similar assessments (i.e., 20 items), it is well suited for gathering data from a large nationally representative sample of teachers. Finally, we chose this measure in line with Lindström and McFadde's (2025) assertion that use of unified materials across populations facilitates a more inclusive approach to advancing reading instruction for students with disabilities. In the following paragraphs, we summarise previous evaluations of psychometric properties of the TKA:SOL, highlighting differences in results obtained using both CTT and IRT.

Bos et al. (2001) developed the TKA:SOL, combining original items with items adapted from instruments used in prior research (Lerner 1997; Moats 1994; Rath 1994), and administered it to a sample of 252 preservice and 286 in‐service teachers. They did not report any procedures for collecting validity evidence based on test content, but they did indicate that they conducted a field test with a small sample (*n* = 55) and reduced the number of items on the initial assessment from 25 to 20. A rationale for item reduction was not specified. For the 20‐item TKA:SOL, they calculated Cronbach's alpha as a measure of internal consistency and reported a value below the acceptable range (α = 0.60; Bandalos 2018), citing the low number of items and low variability as a possible explanation for the result. They treated the measure as unidimensional, despite including items related to two reading domains: phonological awareness and phonics. Finally, they reported item difficulties (i.e., percentage of correct responses) for each item, disaggregated by pre‐ and in‐service teachers. On average, preservice teachers responded correctly to 10.6 questions (53%; SD = 2.8), and in‐service teachers correctly answered 12 (60%; SD = 2.8). Respondents had the lowest mean accuracy on K9—*count phonemes in box* (15%), K6—*voiced‐unvoiced pair* (22%), K15—*define phonics* (30%), K14—*isolate second phoneme in* queen (32%) and K13—*define phonological awareness* (33%).

In a more recent study, Beachy et al. (2023) administered the 20‐item TKA:SOL as part of a larger survey with four modular components. First, they reported results from a sample of 178 PK‐12 teachers from 13 states (60% from Texas) with a goal of refining the measure. Via exploratory factor analysis (EFA), the authors identified two subscales: phonological awareness and phonics. They reported using IRT to calculate item difficulty and discrimination. Though procedures for calculating these values were not specified, the authors reported an overall item difficulty of 66.39 and a discrimination index of 0.39 for all 20 items. Using multiple criteria (i.e., Cronbach's alpha, EFA, discrimination index), they deleted five items: K5—*define consonant blend*, K12—*phoneme blending*, K13—*define phonological awareness*, K15—*define phonics* and K17—*matching phonemes*.

Then, Beachy et al. (2023) administered the reduced 15‐item assessment to a sample of teachers from one school district in Texas (*n* = 91). Results of a confirmatory factor analysis indicated good or adequate model fit statistics for the two‐factor model: phonological awareness (α = 0.558) and phonics (α = 0.565). Further, discrimination index values were ≥ 0.30 for all items, indicating good discriminability. Of the 15 items, teachers correctly answered an average of 63.9% of questions, which represents a slight increase over Bos et al.'s (2001) report of 60% average accuracy on the full measure.

### 1.2 Relations to Other Variables

Prior studies have tested associations between teachers' foundational reading knowledge and three variables: years of teaching experience, degree status and proportion of instructional time spent on foundational reading. A positive and significant association has been reported between foundational reading content knowledge and teaching experience, with the latter coded as a continuous (Piasta et al. 2009) or categorical variable (Beachy et al. 2023; Bos et al. 2001). In contrast, two studies including degree status as a variable of interest (Beachy et al. 2023; Piasta et al. 2009) reported no significant differences in knowledge for teachers with, without or working toward a master's degree. Finally, Piasta and colleagues computed the time teachers spent on explicit phonics instruction during classroom observations. Though teachers' knowledge scores did not predict the amount of time they spent on phonics, students' reading achievement increased as teachers with high levels of foundational reading knowledge provided more minutes of explicit phonics instruction. In each of these prior studies, associations between teachers' *total* knowledge score and their experience, degree and time use were tested. In this study, we evaluate differences in teachers' knowledge at the *item* level based on these three characteristics.

### 1.3 Purpose and Research Questions

The purpose of this study is threefold. We aim to descriptively report knowledge of foundational reading among SET‐IDDs, evaluate the psychometric properties of Bos et al.'s (2001) *Teacher Knowledge Assessment: Structure of Language* (TKA‐SOL) and examine DIF based on teacher characteristics. We aimed to address the following research questions:

1. How well can special education teachers of students with IDD **(**SET‐IDDs) answer questions assessing their foundational word reading content knowledge?
2. What are the psychometric properties of the TKA‐SOL for SET‐IDDs, specifically in terms of person and item reliabilities, as well as the item difficulty relative to each other and to the targeted population?
3. Are there item‐level differences in SET‐IDDs' knowledge based on their teaching experience, highest earned degree and self‐reported time spent teaching phonics and phonemic awareness?

## 2 Method

### 2.1 Participants and Sampling

We recruited a nationally representative sample of special education teachers (*N* = 337) from 41 states. Eligible teachers had to: (a) be a practising special education teacher; (b) teach at least one student with IDD (i.e., autism, intellectual disability, developmental disability and other health impairment); and (c) provide reading instruction to their students. We used *The Generalizer* webtool (Tipton and Miller 2024) to generate four lists of schools, stratified across demographic characteristics (e.g., school size and race/ethnicity). Beginning at the top of each list, research assistants collected teachers' email addresses from faculty directories on school websites, where available. Secondary recruitment efforts included dissemination of advertisements through related US professional organisations' (i.e., Council for Exceptional Children; American Association on Intellectual and Developmental Disabilities) research conferences and newsletters. We required potential respondents to provide a school email address which we used to verify their eligibility prior to sharing a survey invitation.

Teachers were primarily White (83.7%) and female (93.2%), with an average of 13.23 (SD = 9.35) total years of teaching experience. Approximately two‐thirds of participants earned a master's degree or above. They reported teaching in schools across all five US regions, with the largest proportion of participants teaching in the Northeast (42.7%) and West (20.5%). All respondents taught at least one student in Grades K–5, but a small proportion of teachers also taught students in prekindergarten (6.2%) or Grades 6–12 (16.3%). Teachers taught in resource rooms (51%), inclusive classrooms (28.5%) or self‐contained cross‐categorical classrooms (24.9%). In line with inclusion criteria, teachers reported working with students with autism (90.8%), intellectual disability (70.9%), multiple disabilities (35.9%) and/or developmental delay (1.8%). Additionally, many teachers also reported working with students with specific learning disability (72.7%), or speech or language impairment (58.2%). Additional teacher characteristics are reported in Table 1, and a summary of teachers' classroom contexts is reported in Table 2.

**TABLE 1:** Teacher demographics and characteristics.

Characteristic	*n*		*%*
Gender	337		—
Female	314		93.2
Male	13		3.9
Nonbinary	2		0.6
No response	8		2.4
Race/ethnicity	337		—
Black/African American	21		6.2
White	282		83.7
Hispanic or Latinx	13		3.9
Asian or Pacific Islander	9		2.7
Multiracial	3		0.9
Native American/Alaska Native	2		0.6
Not listed	1		0.3
No response	8		2.4
United States region	337		—
Northeast (CT, MA, MD, ME, NH, NJ, NY, PA, RI, VT)	144		42.7
Midwest (IA, IL, IN, KS, MI, MN, MO, ND, NE, OH, SD, WI)	35		10.4
Southeast (AL, AR, DE, GA, FL, KY, LA, MS, NC, SC, TN, VA, WV)	54		16.0
Southwest (AZ, NM, OK, TX)	35		10.4
West (AK, CA, CO, HI, ID, MT, NV, OR, UT, WA, WY)	69		20.5
	*n*	%	TKA:SOL *M* (SD)
Teaching experience (years)			
Special education	334	—	11.16 (8.60)
Total	334	—	13.23 (9.35)
Highest degree earned			
Some college	1	0.3	16.00 (3.39)
Bachelor's	113	33.5	11.65 (3.14)
Master's	214	63.5	12.05 (7.57)
Professional	3	0.9	8.67 (7.57)
Doctorate	6	1.8	13.50 (1.64)
Time spent teaching PA/phonics (%)	219	—	35.03 (20.13)

*Note:* Totals for race/ethnicity exceed the total number of responses because respondents could select all that applied.

Abbreviation: PA, phonemic awareness.

**TABLE 2:** Teacher context.

Variable	*n*	*%*
Grade level	337	—
Prekindergarten	21	6.2
Kindergarten	158	46.9
First	173	51.3
Second	188	55.8
Third	214	63.5
Fourth	210	62.3
Fifth	182	54.0
Sixth	51	15.1
Seventh	18	5.3
Eighth	21	6.2
Ninth	6	1.8
Tenth	4	1.2
Eleventh	4	1.2
Twelfth	6	1.8
Teaching environment	337	—
Resource classroom in a public school	172	51.0
Inclusive classroom in a public school	96	28.5
Self‐contained cross‐categorical classroom in a public school	84	24.9
Self‐contained disability‐specific classroom in a public school	48	14.2
Homebound, going to students' homes	4	1.2
Separate public school for students with disabilities	1	0.3
Alternative private/public school for students removed from other schools	0	0.0
Not listed	7	2.1
Students' primary disabilities	337	—
Autism	306	90.8
Intellectual disability	239	70.9
Specific learning disability	245	72.7
Speech or language impairment	196	58.2
Emotional disturbance/behaviour disorder	135	40.1
Multiple disabilities	121	35.9
Orthopaedic impairment	40	11.9
Other health impairment	54	16.0
Visual impairment	39	11.6
Hearing impairment	35	10.4
Traumatic brain injury	24	7.1
Developmental delay	6	1.8
Not listed	3	0.9

*Note:* Totals for grade level, teaching environment and students' primary disabilities exceed the total number of responses because respondents could select all that applied.

### 2.2 Foundational Reading Knowledge Assessment

Bos et al.'s (2001) *Teacher Knowledge Assessment: Structure of Language* was designed to assess elementary teachers' knowledge of phonological awareness and phonics. Their instrument includes 20 multiple‐choice items, each with four to five response options. Some items assess respondents' knowledge of definitions (e.g., *A pronounceable group of letters containing a vowel sound is a …?)* and others require respondents to apply their knowledge of a language concept (e.g., *If* tife *were a word, the letter* i *would probably sound like the* i *in …?*). We adapted the measure to include an ‘I don't know’ answer choice for each item, similar to the ‘no idea’ option used by Binks‐Cantrell et al. (2012). We made this change after a field test respondent raised the issue of guessing. The full survey is available in supplementary materials (see Appendix S1).

### 2.3 Procedures

#### 2.3.1 Data Integrity

Because we collected survey responses via an online platform, we used several tools and strategies to protect our data quality (Meade and Craig 2012; Roberts and Allen 2015). We distributed the survey through the REDCap web platform (Harris et al. 2009) and enabled security features including a secure, invitation‐only survey form with reCAPTCHA. We embedded an attention check (i.e., repeating the question ‘In which U.S. state do you currently teach?’) and three trap questions (e.g., ‘… what is the fourth month of the year?’). After exporting data from REDCap, we analysed the quality of survey responses, including the respondents' survey completion time. We excluded responses violating any of the following criteria: (a) failed attention check or trap question (*n* = 5); (b) did not teach an eligible grade level (*n* = 21) or student population (*n* = 11); and (c) completed the survey in under 10 min (*n* = 3).

#### 2.3.2 Generalisability

Using *The Generalizer* webtool (Tipton and Miller 2024), we uploaded a list of each school's identification number from the National Center for Education Statistics (NCES) and calculated a generalisability index value for the United States. Values range from 0 to 1, with values greater than or equal to 0.9 indicating high generalisability, in this case to the United States (Tipton and Olsen 2018). Our sample is highly generalisable, as indicated by a generalisability index value of 0.97 (Tipton and Miller 2024).

### 2.4 Data Preparation

To prepare our data for analyses, we scored each TKA:SOL item dichotomously (i.e., 1, *Correct*; 0, *Incorrect*) and examined missing data patterns in SPSS version 29. Responses of ‘I don't know’ and missing data values were scored as incorrect. We calculated a total knowledge score by summing all item responses. Additionally, to prepare data for DIF analyses, we drew on previous research to recode three dichotomous teacher characteristic variables: years of experience (i.e., ≤ 5 years, > 5 years), highest earned degree (i.e., bachelor's and below vs. master's degree and beyond) and time spent on phonemic awareness and phonics (i.e., ≤ 20%, > 20%). Frequencies for each dichotomous variable used in DIF are reported in Table 3. Cutoff scores for level of experience reflected research indicating a shift in Year 5, before which up to half of teachers leave the profession (Cells et al. 2023; Tricarico et al. 2014). Cutoff scores for time spent on foundational reading align with median time spent on these skills in previous observation studies (e.g., Lindström and Lemons 2021).

**TABLE 3:** Dichotomous variables for DIF analysis.

Characteristic	*n*	*%*	TKA:SOL *M* (SD)
Total teaching experience			
≤ 5 years	80	23.7	12.13 (3.18)
	254	75.4	11.25 (3.49)
No response	3	0.9	—
Highest degree earned			
Bachelor's or below	114	33.8	11.96 (3.18)
Master's and above	223	66.2	11.90 (3.32)
Time spent teaching PA/phonics			
≤ 20%	66	19.6	10.97 (3.49)
> 20%	153	45.4	12.58 (2.96)
Missing data	118	35.0	11.59 (3.36)

*Note: N* = 337; PA = phonemic awareness.

### 2.5 Data Analysis

We used the Rasch model (Rasch 1960) to analyse dichotomously scored knowledge survey data. This model is represented by the equation ln[P_ij_/(1‐P_ij_)] = *θ*_*i*_ *—b*_*j*_. In this model, the parameter *θ*_*j*_ represents the latent ability for person *i*, and parameter *b*_*j*_ represents the difficulty of item *j* where the item location on the *X*‐axis (for *θ*) corresponding to the probability of getting the item correct is equal to 0.50. We conducted our analyses in WINSTEPS version 5.7.1.0 (Linacre 2024), using the joint maximum likelihood estimation with all data available. We first examined the output regarding dimensionality of the data, and results indicated that the assumption of unidimensionality was met, with a first contrast eigenvalue of 1.87. We then examined unstandardised mean squares (MNSQ) fit statistics for each item and applied a criterion that MNSQ values between 0.7 and 1.3 be acceptable for our dichotomous test data; items with values exceeding 1.3 indicate underfit and are to be removed (Linacre 2002). Next, we reran the Rasch analysis for the entire scale with misfitting items removed. We reported Rasch reliability estimates for persons and items and further examined the Wright item‐person map for the order of item difficulty along the distribution of respondents.

#### 2.5.1 DIF

DIF contrast sizes can be used to detect differential item function across subgroups (Bandalos 2018). As part of the Rasch analysis, we examined three dichotomous variables of interest for DIF analyses: (a) teacher's level of experience (i.e., ≤ 5 years vs. > 5 years); (b) teachers' highest earned degree (i.e., bachelor's and below vs. master's degree and beyond); and (c) the summed percentage of reading instructional time teachers reported spending on phonemic awareness and phonics (i.e., ≤ 20% vs. > 20%). We examined DIF contrast values and used Zwick et al.'s (1999) recommendation to flag items with values ≥ |0.638| as moderate to large, and those between 0.426 and 0.638 as slight to moderate.

## 3 Results

### 3.1 Descriptive Statistics

Teachers scored an average of 13.2 (66.0%; SD = 3.5) items correct on the full 20‐item measure and 11.9 (66.1%; SD = 3.3) on the reduced 18‐item measure. Response selection frequencies for each item are reported in Table 4. For eight items, some portion of respondents (range: 2–70) selected ‘I don't know’. The greatest number of respondents indicated that they did not know the answer to K6—*voiced‐unvoiced pair*. All 20 items on the TKA:SOL had missing data values (between 2 and 7 missing values per item). Of the 337 survey responses, 27 had missing data (between 1 and 19 missing values per respondent).

**TABLE 4:** Response selection frequencies.

Item ID	Item descriptor	Option A	Option B	Option C	Option D	Option E	Option F	Missing	% Correct
K1	Identify word with short vowel sound	4	8	**311**	4	1	6	3	93.1
K2	Define phoneme	10	**302**	16	2	5	N/A	2	90.1
K3	Define syllable	20	37	**216**	33	26	N/A	5	65.1
K4	Identify word with long vowel sound	13	4	**309**	2	1	4	4	92.8
K5	Define consonant blend	0	43	15	**247**	25	N/A	7	74.8
K6	Identify voiced‐unvoiced pair	14	**114**	40	40	50	70	9	34.8
K7	Define digraph	12	29	0	**257**	21	12	6	77.6
K8	Count phonemes (eight)	**292**	28	3	1	6	N/A	7	88.5
K9	Count phonemes (box)	15	21	247	**47**	2	N/A	5	14.2
K10	Count phonemes (grass)	17	106	**203**	2	6	N/A	3	60.8
K11	Identify example of phoneme deletion	2	3	48	**275**	5	N/A	4	82.6
K12	Identify example of phoneme blending	**285**	0	41	1	5	N/A	5	85.8
K13	Define phonological awareness	58	55	**128**	46	43	N/A	7	38.8
K14	Isolate the second phoneme (queen)	4	248	1	**79**	2	N/A	3	23.7
K15	Define phonics	**209**	30	18	21	38	17	4	62.8
K16	Identify example of soft c	8	16	1	**296**	3	7	6	89.4
K17	Identify two words that begin with the same phoneme	9	**295**	2	17	9	N/A	5	88.9
K18	Reverse order of phonemes (ice)	65	15	9	**229**	14	N/A	5	69.0
K19	Reverse order of phonemes (enough)	25	38	**225**	3	41	N/A	5	67.8
K20	Identify example with no silent letters	52	50	56	10	**126**	38	5	38.0

*Note: N* = 337. Percentage of items correct is reported as the valid percentage. Bold font indicates the correct answer choice. Response options for each item are available in Appendix S1. Percent correct reflects the valid percentage for each item.

### 3.2 Rasch Fit Statistics and Item Difficulty

In the first Rasch analysis (i.e., using all 20 items), we identified two items that did not meet item fit criteria (K2 and K13), so we dropped them, one at a time, and reran the Rasch analysis. All other 18 items had acceptable MNSQ values (i.e., there were no more misfitting items). Item difficulty values ranged from −2.11 to 3.57 logits. Average item difficulty in WINSTEPS is set at 0.00 by default (i.e., 50% chance of selecting the correct answer; see Table 5).

**TABLE 5:** Rasch item difficulty, infit and outfit mean square statistics.

Item ID	Item descriptor	Difficulty measure	S.E.	Infit MNSQ	Outfit MNSQ
K1	Identify word with short vowel sound	−2.11	0.23	1.01	0.86
K3	Define syllable	0.31	0.13	1.00	0.98
K4	Identify word with long vowel sound	−2.06	0.23	0.93	0.67
K5	Define consonant blend	−0.30	0.14	0.87	0.76
K6	Identify voiced‐unvoiced pair	1.97	0.13	1.10	1.18
K7	Define digraph	−0.48	0.15	0.88	0.84
K8	Count phonemes (eight)	−1.45	0.19	1.02	0.97
K9	Count phonemes (box)	3.57	0.19	0.98	0.90
K10	Count phonemes (grass)	0.54	0.13	0.90	0.91
K11	Identify example of phoneme deletion	−0.87	0.16	1.07	1.11
K12	Identify example of phoneme blending	−1.16	0.17	1.10	1.10
K14	Isolate the second phoneme (queen)	2.69	0.15	1.07	1.30
K15	Define phonics	0.43	0.13	1.12	1.25
K16	Identify example of soft c	−1.57	0.19	1.09	1.02
K17	Identify two words that begin with the same phoneme	−1.49	0.19	0.95	0.99
K18	Reverse order of phonemes (ice)	0.08	0.13	0.95	0.90
K19	Reverse order of phonemes (enough)	0.15	0.13	0.91	0.80
K20	Identify example with no silent letters	1.77	0.13	1.01	1.06
	M	0.00	0.16	1.00	0.98
	SD	1.59	0.03	0.08	0.16

*Note:* MNSQ = unstandardised item fit statistics.

### 3.3 Rasch Person and Item Reliabilities

Rasch person reliability was 0.68 for all respondents and 0.63 excluding extreme respondents (i.e., excluding highest and lowest possible ratings for all items). Person reliability was slightly low (< 0.80), indicating low to moderate variability among respondents and/or a need to add more distinguishing items (Linacre Forthcoming). Item reliability was 0.99, suggesting items are well spread out, and their ranking is consistent (Linacre 1997). Additional reliability and separation indices are reported in Table 6.

**TABLE 6:** Rasch mean measure and SD, Rasch reliability and separation indices and classical reliability (18 knowledge items).

	Person (all sample)	Person (nonextreme)	Item
Mean measure	1.14	1.05	0.00
SD	1.18	0.95	1.58
Separation	1.46	1.30	9.42
Rasch reliability	0.68	0.63	0.99
Cronbach's α (S.E.)	0.75 (1.59)		

Abbreviation: S. E., standard error.

### 3.4 Rasch Item‐Person Variable Maps

**FIGURE 1:**
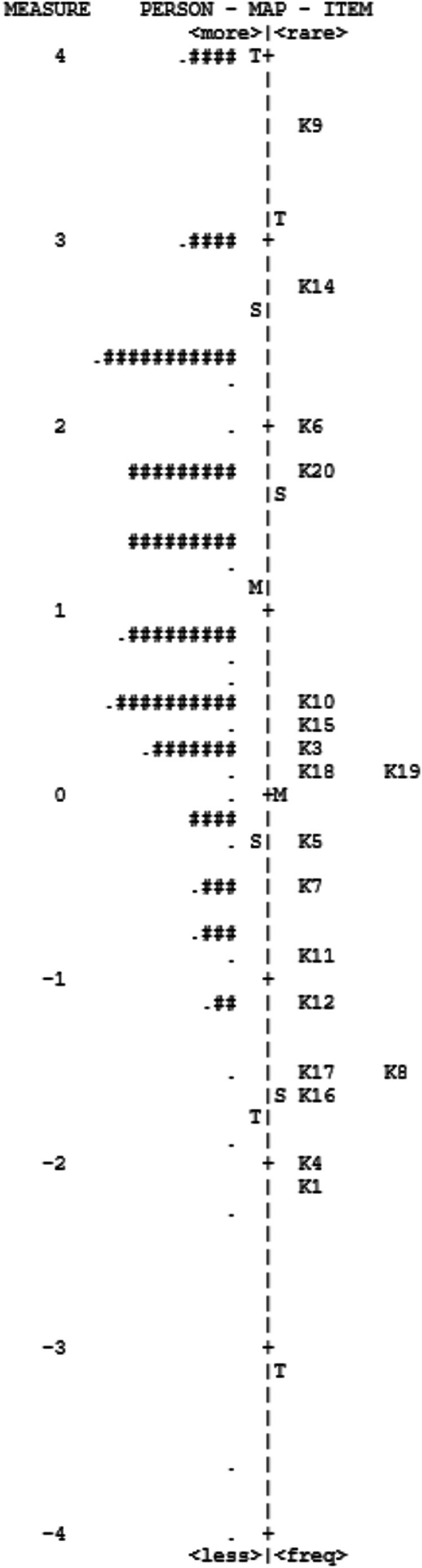
Rasch Wright Person‐item map. Each *#* is four respondents; each ‘.’ represents one to three respondents.

In Figure 1, we display the item‐person variable map. This map shows along the same logit scale the distribution of participants and the hierarchy of item difficulty, from the most difficult item (top) to the least difficult (bottom). The most difficult item was K9—*count phonemes in* box, and the easiest was K1—*short vowel sound*.

### 3.5 DIF

#### 3.5.1 Teaching Experience DIF

DIF values for teaching experience (i.e., > 5 years vs. ≤ 5 years) ranged from |+0.47|to|−0.71|. We flagged five items with slight to moderate effect sizes (| ±0.426|logits < ES <| ±0.638|logits) and three items with moderate to large effect sizes (ES > |±0.638|logits). Teachers with more than 5 years of teaching experience found two items slightly to moderately more difficult (i.e., K6—*identify voiced‐unvoiced pair*, K20—*identify example with no silent letters*) than teachers with less experience. Both items had above‐average difficulty, relative to all items on the assessment.

Teachers with more experience found three items slightly to moderately easier than teachers with less experience (i.e., K1—*short vowel sound,* K12—*identify example of phoneme blending* and K16—*soft c*) and they found three items much easier (i.e., K8—*count phonemes in eight*, K9—*count phonemes in* box and K15—*define phonics*). For the entire sample, some of these had above‐average difficulty (i.e., K9, K15), and others had below‐average difficulty (i.e., K1, K8, K12, K16). Item K9 was most difficult for all respondents and separated teachers with more and less teaching experience to the greatest extent. We observed negligible DIF contrasts (i.e., <| ± 0.426|) for the remaining 10 items.

#### 3.5.2 Degree Status DIF

DIF contrast values for degree status (i.e., bachelor's or below, advanced) ranged from +0.71 to −0.87. We detected a slight to moderate contrast for one item (i.e., K15—*define phonics*) and moderate to large contrasts for two items (i.e., K16—*soft c;* K18—*reverse phonemes in* ice). Teachers with advanced degrees found K15 and K16 (and K18 more difficult) easier than teachers with bachelor's degrees or below. Item K16 had below‐average difficulty, but K15 and K18 were slightly more difficult than average, relative to all items for the full sample.

#### 3.5.3 Time Spent Teaching Phonemic Awareness and Phonics DIF

The third variable for which we examined DIF was the percentage of reading instructional time teachers reported spending on phonemic awareness and phonics (i.e., ≤ 20% vs. > 20%). We detected slight to moderate contrasts for five items (i.e., K8—*count phonemes in* eight, K11—*phoneme deletion*, K17—*matching phonemes*, K19—*reverse order of phonemes in* enough and K20—*silent letters*) and moderate to large contrasts for three items (i.e., K1—*short vowel sound*, K4—*long vowel sound* and K10—*count phonemes in* grass). Teachers who spent more than 20% of instructional time on phonemic awareness and phonics found K1, K8, K10 and K11 easier than teachers spending less time on phonics and phonemic awareness instruction. Notably, K1, K8 and K11 were among the easier items on the assessment overall, but K10 was slightly more difficult than average. Teachers who spent more time on phonics and phonemic awareness found K4, K17, K19 and K20 more difficult than those spending less time in these areas. K4 and K17 were also among the easier items on the assessment, and K19 and K20 were more difficult than average. Contrasts for each item are available in Table 7.

**TABLE 7:** DIF contrasts for teacher experience, degree earned and time spent on pa and Phonics.

Item	Item descriptor	Experience (> 5 years–≤ 5 years)		Degree (Adv—Bach)		PA/phonics time (> 20%–≤ 20%)	
		Contrast	Joint S.E.	Contrast	Joint S.E.	Contrast	Joint S.E.
K1	Identify word with short vowel sound	−0.48*	0.49	−0.17	0.48	−0.75**	0.65
K3	Define syllable	0.40	0.31	0.16	0.28	−0.41	0.35
K4	Identify word with long vowel sound	0.03	0.52	0.16	0.49	1.03**	0.82
K5	Define consonant blend	0.04	0.33	0.19	0.3	0.26	0.37
K6	Identify voiced‐unvoiced pair	0.47*	0.32	−0.02	0.29	−0.03	0.37
K7	Define digraph	−0.16	0.33	−0.14	0.31	0.38	0.39
K8	Count phonemes (eight)	−0.65**	0.40	−0.05	0.39	−0.46*	0.46
K9	Count phonemes (box)	−0.67**	0.57	−0.27	0.42	0.02	0.53
K10	Count phonemes (grass)	0.37	0.30	0.25	0.27	−1.10**	0.35
K11	Identify example of phoneme deletion	−0.09	0.36	−0.16	0.33	−0.60*	0.41
K12	Identify example of phoneme blending	−0.44*	0.37	−0.21	0.36	−0.03	0.48
K14	Isolate the second phoneme (queen)	0.14	0.37	−0.11	0.32	−0.11	0.40
K15	Define phonics	−0.71**	0.30	−0.47*	0.27	0.38	0.35
K16	Identify example of soft c	−0.45*	0.41	−0.87**	0.39	0.16	0.52
K17	Identify two words that begin with the same phoneme	−0.04	0.42	0.00	0.4	0.58*	0.50
K18	Reverse order of phonemes (ice)	0.29	0.32	0.71**	0.3	0.02	0.36
K19	Reverse order of phonemes (enough)	0.19	0.31	0.31	0.28	0.47*	0.37
K20	Identify example with no silent letters	0.46*	0.31	0.00	0.28	0.49*	0.35

*Note:* * = slight to moderate effect size (| ± 0.426|logits < ES < | ± 0.638| logits); ** = moderate to large effect size (ES > | ± 0.638|logits).

Abbreviations: Adv, master's degree and beyond; Bach, bachelor's degree or below.

## 4 Discussion

In this study, we aimed to measure SET‐IDDs' foundational reading knowledge and examine differences in SET‐IDDs' knowledge at the item level based on three teacher characteristics: experience, degree status and self‐reported instructional time spent on foundational reading skills. Overall, teachers correctly answered approximately two‐thirds of the TKA‐SOL items. Interestingly, we did not detect a consistent pattern in item‐level accuracy based on teachers' characteristics, such that teachers with greater experience, training and time spent on foundational reading both outperformed and underperformed those with less of each trait. The following section connects our findings with prior evaluations of teachers' foundational reading knowledge.

### 4.1 Connections to Previous Research

Our results align with findings of prior studies of teachers' knowledge of reading, which have suggested that teachers are lacking foundational reading content knowledge (e.g., Bos et al. 2001; Moats 1994). The mean total score for our sample, which only included special education teachers, was 13.2 out of 20 (66%) points and 11.9 out of 18 (66.1%) points in our final model. When they administered the TKA:SOL, Bos et al. (2001) reported an average score of 10.6 out of 20 points (53%) for preservice teachers and 12.0 out of 20 (60%) for in‐service teachers. In‐service special education teachers comprised 31% of their sample, and they scored slightly higher on the TKA:SOL (M = 12.7; 63.5%) than in‐service general education elementary teachers (M = 12.0; 60%). Using a reduced version of the same measure, Beachy et al. (2023) reported a mean score of 63.9%. Our findings indicate alignment with those of Bos et al. and Beachy et al., despite differences in sampling (i.e., three universities in two US regions or one district in Texas vs. national sample). Although our focus is on SET‐IDDs, one possible explanation for this alignment may be owed to 73% of respondents in our sample reporting that they also teach students with specific learning disabilities and thus likely having greater access to pre‐ and in‐service training on reading instruction than instructors who solely teach students with IDD (National Council on Teacher Quality 2020).

A comparison of item difficulties between our results and previous studies (Beachy et al. 2023; Bos et al. 2001) revealed that the most difficult item (K9—*count phonemes in* box) remained the same for all three samples. Our participants' mean item scores were higher for 13 of the 20 items (M difference = 9.03%; range: 0.1%–32.8%) compared to Bos and colleagues' sample, with the most substantial difference noted for K15—*define phonics*. Further, teachers in our sample scored higher on all six items testing knowledge of terminology (i.e., definitions items). In contrast, Beachy and colleagues' national sample performed better than ours on a majority of items (11 of 20; *M* difference = 8.30; range: 0.2%–29.4%). The largest contrast was for item K20—*identify an example with no silent letters*, as nearly 30% more of their participants responded correctly than our respondents. In short, our sample performed better than Bos and colleagues' on most items but scored lower than Beachy and colleagues' sample on a slight majority of items. Considered together, these findings indicate that SET‐IDDs have less knowledge about foundational reading than a recent broader sample of special education teachers (i.e., teachers that do and do not teach students with IDD), with possible knowledge gaps (> 10% difference) related to silent letters and isolating/matching phonemes.

### 4.2 DIF Analyses

The results of our DIF analyses demonstrated inconsistencies in teachers' performance based on their level of teaching experience (i.e., ≤ 5 vs. > 5 years), degree status (i.e., bachelor's or below vs. advanced) and self‐reported time spent teaching phonemic awareness and phonics (i.e., ≤ 20% vs. > 20%), such that some items were both more difficult and less difficult for members of each group. Due to the inconsistencies in teachers' performance based on group membership, we can only conclude that teachers with varying amounts of experience, training and time spent on phonics hold different sets of knowledge. These findings may reflect varied approaches to teacher training at the national level, such that universities and alternative training programmes may differ vastly in their course offerings, practicum experiences and other requirements related to reading methods (Schwartz 2024).

Our findings underscore questions about pre‐ and in‐service preparation of SET‐IDDs in reading foundations. In their survey of 147 educators in the United States who teach reading to students with IDD, Conner et al. (2022) found that 23%–36% of respondents reported not receiving training at their university in one or more areas of literacy (e.g., phonics and vocabulary) and half of respondents or more received no in‐service training on these topics. Regarding self‐efficacy, most respondents (56.5%) did not agree very strongly that they had the knowledge required to teach reading to all of their students. Although the TKA:SOL does not have guidelines for interpreting teacher proficiency, it provides important information about teachers' subject matter content knowledge (Shulman 1987), which complements instructional skill (Brownell et al. 2020).

Our findings pertaining to years of experience and level of degree may be explained in part by those from the National Center for Teaching Quality (2020). Although their findings indicate overall growth in emphasis on reading foundations in teacher preparation programmes in the last 12 years, their findings also indicated a discrepancy between undergraduate and graduate programmes, such that the former have more stagnant growth in this area since 2016. Additionally, our results do not indicate a recency effect in which teachers with less experience demonstrate higher knowledge than those with more experience. This pattern may reflect contributions of in‐service training, whether mandated by state policy as in 38 of 50 states (Schwartz 2024) or prioritised by individual districts and schools. It should be noted that in both reports, findings pertain to elementary teacher preparation programmes at large, and they do not report disaggregated data for special education teachers in general nor those working specifically with students with IDD. Future research is needed to better understand the nature of reading content in teacher preparation programmes and in‐service professional development for SET‐IDDs in particular.

### 4.3 Limitations

There are several limitations that should be considered when interpreting the results of this study. First, because we recruited a national sample of teachers and administered the knowledge assessment via an online survey, it is possible that some invalid responses went undetected. Though we used an invitation‐only recruitment procedure using respondents' school email addresses, it is possible that respondents did not adequately attend to questions or that they did not provide accurate responses regarding their eligibility to participate.

Second, we adapted Bos et al.'s (2001) measure and added an ‘I don't know’ option to each knowledge item. It is possible that respondents with the same level of knowledge of language could have earned different scores on the assessment due to differences in their propensity for guessing (Mondak 2001). However, a small proportion of respondents selected the ‘I do not know’ option for most items.

Third, we recoded three grouping variables to meet the requirements of DIF analysis: experience, highest degree earned and instructional time use. Some precision is lost when continuous variables are recoded into categorical variables, as we did for years of experience and instructional time use. Additionally, we merged participant groups for degree status prior to conducting DIF analyses. Sample sizes for the professional (*n* = 3) and doctorate (*n* = 6) were too small for trustworthy DIF analysis; thus, we merged these more advanced degrees with the master's degree category. As the sample size was too small to permit inferential analyses, we only reported descriptive statistics (see Tables 1 and 3) for total knowledge score for each degree status group. Some nuance in knowledge differences between teachers with master's and doctoral degrees may not have been captured, as those with doctoral degrees had a higher mean score on the TKA:SOL.

Finally, about two‐thirds of respondents did not accurately interpret the directions for self‐reporting their time use during reading instruction. Some participants reported minutes spent in each category rather than the percent of total reading instructional time. As such, we dropped approximately one‐third of responses for which total self‐reported time use across areas of reading did not equal 100%. This reduced the sample size for the DIF analysis.

### 4.4 Future Directions for Research

Teacher knowledge of foundational reading is important because of its potential connection to classroom instruction and student growth (see Piasta et al. 2009) and its malleability (i.e., as a target for pre‐ and in‐service teacher training). To better understand this construct in the context of instruction for students with IDD, future studies may explore the connection of SET‐IDDs' foundational reading knowledge with their self‐efficacy and beliefs in this area (see Conner et al. 2022). Additionally, connecting teachers' knowledge to their classroom practices via observation and/or other descriptive studies may provide important information about how training is enacted in instruction. Furthermore, as students with IDD are likely to receive instruction from professionals other than their lead classroom teacher (e.g., paraeducators and speech‐language pathologists; Lindström et al. 2023), future studies may also examine these individuals' knowledge in foundational reading.

Finally, as governments in the United States and internationally implement policies addressing pre‐ and in‐service teachers' training to support students with reading difficulties (Maxwell 2019; Youman and Mather 2015), research is needed to understand how these policies influence reading instruction for students with IDD. Analysis of policy language may be valuable in understanding the extent to which students with IDD are considered in the development and implementation of such policies.

### 4.5 Implications for Practice

The inconsistencies in SET‐IDDs' knowledge, regardless of their degree status, experience and percentage of instructional time dedicated to phonemic awareness and phonics, suggest that teacher preparation programmes and in‐service training are not adequately meeting teachers' needs. Although our sample performed slightly better than that of Bos et al. (2001), such a difference between samples does not seem practically meaningful in the quarter century since. In‐service special education teachers, including SET‐IDDs, need training and ongoing support and feedback to facilitate their development of foundational reading content knowledge. Given our finding in which years of experience did not directly predict respondents' accuracy, schools may consider professional development and peer mentoring models that are less dependent on teaching experience and prioritise depth of knowledge.

Additionally, preservice teachers preparing to work with students with IDD require more robust training in language to support their ability to teach foundational reading skills effectively, especially given the variability in language skills for children with IDD (Sermier Dessemontet et al. 2017). For example, special education faculty may consider course planning collaboratively with colleagues specialising in communication disorders. They may also evaluate the extent to which academic methods courses address the needs of students with IDD directly, rather than as footnotes in courses focused on students with higher incidence disabilities (Copeland et al. 2011). Finally, as efforts intensify to address teacher knowledge of reading instruction for other populations (e.g., dyslexia), we remind stakeholders to consider implications for all students, especially those with IDD who have been historically excluded from such efforts.

## Ethics Statement

This study was approved by the Institutional Review Board of Lehigh University in Bethlehem, Pennsylvania, US.

## Conflicts of Interest

The authors declare no conflicts of interest.

## Supporting information


**Appendix S1:** Supporting information.

## Data Availability

Available upon request.
