# Supplementary material for: Foundational Reading Knowledge of Teachers of Students With IDD: Examining Experience, Degree and Time Use
Source: J Intellect Disabil Res. 2025 Sep 4;69(12):1435–47. doi: 10.1111/jir.70041 (PMC12580473; doi:10.1111/jir.70041)
Supplement: Supplementary file 1 — Appendix S1: Supporting information. [file JIR-69-1435-s001.docx]

**Supplementary Material - Appendix A**

*Teacher Knowledge Assessment: Structure of Language*

(Adapted from Bos et al., 2001)

**1) Which word contains a short vowel sound?**

(a) treat

(b) start

(c) slip

(d) cold

(e) point

(f) I don’t know.

**2) A phoneme refers to:**

(a) a single letter

(b) a single speech sound

(c) a single unit of meaning

(d) a grapheme

(e) I don’t know.

**3) A pronounceable group of letters containing a vowel sound is a:**

(a) phoneme

(b) grapheme

(c) syllable

(d) morpheme

(e) I don’t know.

**4) If *tife* were a word, the letter i would probably sound like the *i* in:**

(a) if

(b) beautiful

(c) find

(d) ceiling

(e) sing

(f) I don’t know.

**5) A combination of two or three consonants pronounced so that each letter keeps its own identity is called a:**

(a) silent consonant

(b) consonant digraph

(c) diphthong

(d) consonant blend

(e) I don’t know.

**6) Example of a voiced and unvoiced consonant pair would be:**

(a) b-d

(b) p-b

(c) t-f

(d) g-j

(e) c-s

(f) I don’t know.

**7) Two combined letters that represent one single speech sound are a:**

(a) schwa

(b) consonant blend

(c) phonetic

(d) digraph

(e) diphthong

(f) I don’t know.

**8) How many speech sounds are in the word "eight"?**

(a) two

(b) three

(c) four

(d) five

(e) I don’t know.

**9) How many speech sounds are in the word "box"?**

(a) one

(b) two

(c) three

(d) four

(e) I don’t know.

**10) How many speech sounds are in the word "grass"?**

(a) two

(b) three

(c) four

(d) five

(e) I don’t know.

**11) What type of task would this be? Say the word "cat." Now say cat without the/c/sound.**

(a) blending

(b) rhyming

(c) segmentation

(d) deletion

(e) I don’t know.

**12) What type of task would this be? "I am going to say some sounds that will make one word when you put them together. What does/sh//oe/say?"**

(a) blending

(b) rhyming

(c) segmentation

(d) manipulation

(e) I don’t know.

**13) Mark the statement that is false:**

(a) Phonological awareness is a precursor to phonics;

(b) Phonological awareness is an oral language activity;

(c) Phonological awareness is a method of reading instruction that begins with individual letters and sounds;

(d) Many children acquire phonological awareness from language activities and reading.

(e) I don’t know.

**14) What is the second sound in the word "queen"?**

(a) u

(b) long e

(c) k

(d) w

(e) I don’t know.

**15) A reading method that focuses on teaching the application of speech sounds to letters is called:**

(a) phonics

(b) phonemics

(c) orthography

(d) phonetics

(e) either a or d

(f) I don’t know.

**16) A soft c is in the word:**

(a) Chicago

(b) cat

(c) chair

(d) city

(e) none of the above

(f) I don’t know.

**17) Identify the pair of words that begins with the same sound,**

(a) joke - goat

(b) chef – shoe

(c) quiet - giant

(d) chip = chemist

(e) I don’t know.

**18) If you say the word, and then reverse the order of the sounds, "ice" would be:**

(a) easy

(b) sea

(c) size

(d) sigh

(e) I don’t know.

**19) If you say the word, and then reverse the order of the sounds, "enough" would be:**

(a) fun

(b) phone

(c) funny

(d) one

(e) I don’t know.

**20) All of the following nonsense words have silent letters, except:**

(a) bamb

(b) wrin

(c) shipe

(d) knam

(e) phop

(f) I don’t know.
